# Cortico-basal ganglia networks dysfunction associated with disease severity in patients with idiopathic blepharospasm

**DOI:** 10.3389/fnins.2023.1159883

**Published:** 2023-03-30

**Authors:** Qinxiu Cheng, Han Xiao, Yuhan Luo, Linchang Zhong, Yaomin Guo, Xinxin Fan, Xiaodong Zhang, Ying Liu, Ai Weng, Zilin Ou, Weixi Zhang, Huawang Wu, Qingmao Hu, Kangqiang Peng, Jinping Xu, Gang Liu

**Affiliations:** ^1^Chinese Academy of Sciences, Institute of Biomedical and Health Engineering, Shenzhen Institutes of Advanced Technology, Shenzhen, China; ^2^Department of Nuclear Medicine, Guangdong Second Provincial General Hospital, Guangzhou, China; ^3^Department of Neurology, National Key Clinical Department and Key Discipline of Neurology, Guangdong Provincial Key Laboratory for Diagnosis and Treatment of Major Neurological Diseases, The First Affiliated Hospital, Sun Yat-sen University, Guangzhou, China; ^4^Department of Medical Imaging, Sun Yat-sen University Cancer Center, State Key Laboratory of Oncology in Southern China, Collaborative Innovation Center for Cancer Medicine, Guangzhou, China; ^5^Guangzhou Huiai Hospital, The Affiliated Brain Hospital of Guangzhou Medical University, Guangzhou, China

**Keywords:** cortico-basal ganglia networks, graph theoretical analysis, hemifacial spasm, idiopathic blepharospasm, resting-state functional magnetic resonance imaging

## Abstract

**Background:**

Structural changes occur in brain regions involved in cortico-basal ganglia networks in idiopathic blepharospasm (iBSP); whether these changes influence the function connectivity patterns of cortico-basal ganglia networks remains largely unknown. Therefore, we aimed to investigate the global integrative state and organization of functional connections of cortico-basal ganglia networks in patients with iBSP.

**Methods:**

Resting-state functional magnetic resonance imaging data and clinical measurements were acquired from 62 patients with iBSP, 62 patients with hemifacial spasm (HFS), and 62 healthy controls (HCs). Topological parameters and functional connections of cortico-basal ganglia networks were evaluated and compared among the three groups. Correlation analyses were performed to explore the relationship between topological parameters and clinical measurements in patients with iBSP.

**Results:**

We found significantly increased global efficiency and decreased shortest path length and clustering coefficient of cortico-basal ganglia networks in patients with iBSP compared with HCs, however, such differences were not observed between patients with HFS and HCs. Further correlation analyses revealed that these parameters were significantly correlated with the severity of iBSP. At the regional level, the functional connectivity between the left orbitofrontal area and left primary somatosensory cortex and between the right anterior part of pallidum and right anterior part of dorsal anterior cingulate cortex was significantly decreased in patients with iBSP and HFS compared with HCs.

**Conclusion:**

Dysfunction of the cortico-basal ganglia networks occurs in patients with iBSP. The altered network metrics of cortico-basal ganglia networks might be served as quantitative markers for evaluation of the severity of iBSP.

## Introduction

Idiopathic blepharospasm (iBSP) is a common sub-type of focal dystonia, which is characterized by bilateral intermittent or sustained spasms of the orbicularis oculi muscle, and commonly associated with psychiatric and cognitive co-morbidities that lead to heterogeneous clinical expressions (Alemán et al., 2009; Defazio et al., 2017). Although immense effort has been dedicated to identify its etiology, the full pathophysiology of iBSP is still not well understood. Hemifacial spasm (HFS) was known to be caused often by vessel compression at the root exit zone of the facial nerve (Tan et al., 2004), which is characterized by unilateral clonic or tonic contractions of facial muscles (Chaudhry et al., 2015). Since it showed similar clinical symptoms with iBSP especially for facial hyperkinetic movements, it was commonly used as a control group to further differentiate changes that occur due to dystonia-specific abnormalities or facial hyperkinetic movements (Guo et al., 2021; Xu et al., 2022).

Traditionally, iBSP has been thought to be related to abnormalities in the basal ganglia (Suzuki et al., 2007; Zoons et al., 2011; Guo et al., 2021). With the development of magnetic resonance imaging (MRI) techniques, structural changes extend to many brain regions involved in the basal ganglia-thalamo-cortical motor circuit in iBSP (Obermann et al., 2007; Suzuki et al., 2011; Horovitz et al., 2012; Worbe et al., 2012; Tomić et al., 2021). Classically, these abnormalities observed outside the basal ganglia have been commonly thought as secondary phenomena due to abnormal motor output/sensory input from excessive movements (Lehéricy et al., 2013). However, in addition to motor circuit, the cortical-basal ganglia networks also included brain regions mainly responding for cognitive and emotional processing, such as posterior cingulate, prefrontal, orbitofrontal area, anterior cingulate, insular, hippocampus, and amygdala. The structural changes in these regions were also found to be associated with iBSP (Obermann et al., 2007; Suzuki et al., 2011; Horovitz et al., 2012; Worbe et al., 2012; Tomić et al., 2021). To better understand the pathophysiology of iBSP, a network model has been put forward in a previous review and was further supported by other reviews (Neychev et al., 2011; Niethammer et al., 2011). They suggested that iBSP is a network disorder associated with structural and functional derangement in a network connecting frontal and parietal cortical regions, basal ganglia, thalamus, the cerebellum and et al., rather than resulted from particular abnormalities in certain brain regions. These suggestions were further supported by results from functional MRI (fMRI) studies in the iBSP. Specially, different fMRI methodologies, such as amplitude of low frequency fluctuations, regional homogeneity analysis, seed-based analysis, voxel-mirrored homotopic connectivity, independent component analysis, and graph theoretical analysis, have been widely applied to explore the functional mechanism underlying iBSP (Zhou et al., 2013; Wei et al., 2018; Jiang et al., 2019). They reported abnormal functional connectivity in various cortical and subcortical regions, as well as within default mode network, sensory motor network, fronto-parietal network, and salience network in patients with iBSP (Huang et al., 2017). These results further in favor for the suggestion that iBSP is a network disorder. Among these methodologies, the graph theoretical analysis is the best way to measure brain functional organization at a brain network level or even at the whole brain connectivity patterns (Smitha et al., 2017). Using this method, iBSP patients showed an abnormal functional network architecture at whole brain level characterized by abnormal expansion or shrinkage of neural communities (Battistella et al., 2017). To our best knowledge, no study has examined cortico-basal ganglia networks using resting-state fMRI (rs-fMRI) despite a large body of evidence pointing toward dysfunction in certain regions of cortico-basal ganglia networks in iBSP (Yang et al., 2013; Jochim et al., 2017; Fang et al., 2021). Therefore, how structural changes could influence the functional patterns of cortico-basal ganglia networks and further related to symptom expression, as well as whether these functional alterations were due to dystonic origin or facial hyperkinetic movements in patients with iBSP remain largely unclear.

In the present study, in accordance with the hypothesis that extensively altered functional connections exist in the large-scale cortico-basal ganglia networks in iBSP, we evaluated the global and local organization of functional connections using graph theoretical analysis of cortico-basal ganglia networks between patients with iBSP and healthy controls (HCs). A group of age-, gender-, and duration-matched patients with HFS was also recruited to determine whether these functional alterations in iBSP are derived from dystonic origin or excessive facial movements. Finally, correlation analyses were performed to explore relationships between topological parameters and clinical measurements (e.g., severity and disease duration of iBSP) in patients with iBSP.

## Materials and methods

### Subjects

Patients were recruited from an outpatient clinic of the First Affiliated Hospital of Sun Yat-sen University. They were diagnosed as adult-onset iBSP or HFS based on the standard criteria by two senior neurologists (G Liu and WX Zhang). The exclusion criteria for iBSP and HFS patients were the same with the following items: (i) aged less than 18 or more than 80; (ii) left handed; (iii) received botulinum toxin (BoNT) injections within 3 months before MRI scanning; (iv) had a family history of movement disorders; (v) reported evidence of stroke and traumatic brain injury, or any conditions which might be cause secondary blepharospasm; (vi) reported evidence of neurological diseases, such as Parkinson’s disease, Alzheimer’s disease, major depressive disorder, and epilepsy; (vii) had a history of exposure to previous neuroleptic medication; (viii) had any conditions that contradicted with cerebral MRI; (ix) show evidence of possible anxiety (Hamilton anxiety rating scale score of > 14); and (x) received oral medications for approximately 24 h before MRI scans. HCs were also recruited using the same exclusion criteria. This study was performed according to the Declaration of Helsinki and was approved by the Ethics Committee of the First Affiliated Hospital of Sun Yat-sen University [(2020)323]. Written informed consent was obtained from all subjects. Finally, 62 patients with iBSP, 62 with HFS (31, left side), and 62 HCs were included in the current study.

### Clinical measurements

The demographics and clinical characteristics, including patients’ age, gender, education, duration of disease, and duration of BoNT injections, were obtained from all patients *via* face-to-face interviews before MRI scanning. The disease severity of patients was evaluated using the 0–4 scale Jankovic Rating Scale (JRS), which includes two subscales that measure severity (0 = None; 1 = Increased blinking only with external stimulus; 2 = Mild but spontaneous eyelid fluttering, but not functionally disabling; 3 = Moderate spasm, mildly incapacitating; and 4 = Severe, incapacitating spasm including eyelid and other facial muscles) and frequency (0 = None; 1 = Slightly increased blinking frequency; 2 = Eyelid fluttering shaking lasting less than 1 s; 3 = Orbicularis oculi muscle spasm lasting more than 1 s with eyes opening more than 50% of awake time; and 4 = Functionally “blind”) of involuntary orbicularis oculi muscle contraction, respectively (Jankovic et al., 2009).

### Data acquisition

All rs-fMRI data were obtained using a 3T Siemens scanner (Tim Trio; Siemens, Erlangen, Germany). Participants were instructed to keep their eyes closed and their heads motionless during scanning. An echo-planar imaging sequence with the following parameters was used: repetition time = 2,000 ms, echo time = 30 ms, flip angle = 90°, acquisition matrix = 64×64; voxel size = 3.4375 mm^3^×3.4375 mm^3^×3 mm^3^, 33 slices, and volumes = 240.

### Data pre-processing

The rs-fMRI data were pre-processed using the Data Processing and Analysis of Brain Imaging (DPABI) toolbox^1^ (Yan et al., 2016). The main steps including: (i) discarding the first 10 volumes to allow for magnetization equilibrium; (ii) realigning to the first volume to account for head motion (subjects showing a maximum displacement of more than 3 mm and an angular motion of more than 3° were removed); (iii) normalizing to 3 mm × 3 mm × 3 mm resolution in the Montreal Neurological Institute (MNI) template; (iv) spatially smoothing with a Gaussian kernel of 6 mm full-width at half maximum; (v) regression out Friston-24 head motion parameters, white matter, and cerebrospinal fluid signals (as regression out global mean signals can lead to spurious resting-state functional correlations and false inferences, they were not regression out in the current study); (vi) filtering with the temporal band-path (0.01–0.1 Hz); and (vii) scrubbing bad images (before two time points and after one time point) which exceed the pre-set criterion (frame displacement < 0.5) for excessive motion.

### Network construction

**FIGURE 1:**
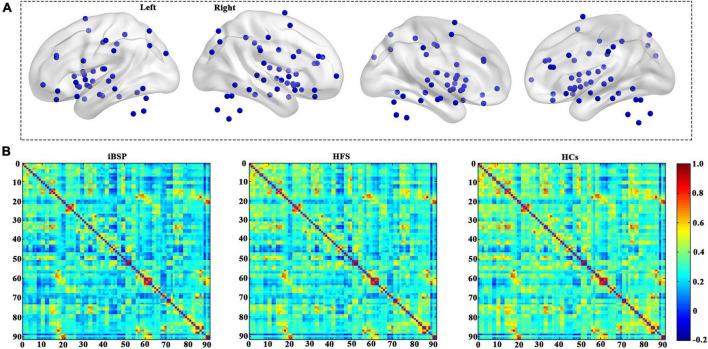
Cortico-basal ganglia networks. **(A)** Distributions of all brain regions in the cortico-basal ganglia networks. All regions are listed in Supplementary Table 1. **(B)** The average functional connectivity matrix of the cortico-basal ganglia networks for patients with idiopathic blepharospasm and hemifacial spasm and HCs. HCs, healthy controls; HFS, hemifacial spasm; iBSP, idiopathic blepharospasm.

To construct the cortico-basal ganglia networks for each participant, we used 91 MNI coordinates that were previously reported (Figure 1A and Supplementary Table 1; Worbe et al., 2012). The node was defined as a sphere with a radius of 6 mm centered at each coordinate and resampled to 3 mm × 3 mm × 3 mm resolution. The functional network edge was defined with resting-state functional connectivity measured using Pearson’s correlation coefficient between the mean time series of each pair of the 91 regions. As a result, a symmetric 91×91 functional connectivity matrix was obtained for each participant for further analyses (Figure 1B). Consistent with previous studies, we only used positive connectivity in the further analysis (Xu et al., 2020; Pang et al., 2022).

### Graph theory-based network analyses

To identify changes in global topological characteristics of the cortico-basal ganglia networks among three groups, global network parameters including global efficiency (E_glob_) that can indicate the efficiency of information transference across a network, clustering coefficients (C_p_) that quantify the local inter-connectivity of a network, and the shortest path length (L_p_) that can quantify the integration of a network, were calculated using a graph theoretical network analysis toolbox (GRETNA v2.0).^2^ E_glob_ and L_p_ are used to measure brain network integration, and C_p_ is employed to investigate brain network segregation. To avoid bias choice of a certain value, the complex network analyses were performed at a sparsity range from 0.1 to 0.5 with an interval of 0.05, and the area under curve (AUC) values under this range of sparsity were calculated for the statistical analyses (Xu et al., 2017). The group difference of network parameters was explored using analysis of covariance (ANCOVA) and *post-hoc* two-sample *t*-tests between any two groups, with age and gender as covariates. The significance level was set at *P* < 0.05.

### Functional connectivity comparisons

To explore group differences in functional connections, an ANCOVA was used to identify differences among the three groups, and two-sample *t*-tests were used to identify differences between any two groups. The results were corrected by Bonferroni corrections of *P* < 1.22×10^–5^.

### Regional indexes comparisons

Moreover, we also calculated the betweenness centrality and degree centrality as regional indexes for each region. To avoid bias choice of a certain value, the complex network analyses were performed at a sparsity range from 0.1 to 0.5 with an interval of 0.05, and the AUC values under this range of sparsity were calculated for the statistical analyses (Xu et al., 2017). The group differences of network parameters and regional indexes were explored using ANCOVA and *post-hoc* two-sample *t*-tests between any two groups, with age and gender as covariates. The significance level was set at *P* < 0.05 for network parameters, and *P* < 0.05/91 (Bonferroni corrections) for regional indexes.

### Correlation with clinical measurements

To reveal whether the changed global metrics and functional connections in the cortico-basal ganglia networks showed associations with clinical measurements in patients with iBSP, partial correlation analyses between changed indices and disease duration and JRS total scores were performed, using age and gender as covariates. The significance level was set at *P* < 0.05.

### Statistical analyses

We analyzed all the subjects’ demographics and clinical characteristics using SPSS 23.0 (IBM, Armonk, NY, USA). Differences in the age among the iBSP, HFS, and HCs groups were identified using the non-parametric Kruskal–Wallis *H* test. Gender differences among the three groups and the number of patients receiving BoNT injections between patients with iBSP and HFS were compared using the Pearson *χ^2^* test. Moreover, differences in the disease duration, JRS total scores, and duration of BoNT injections between patients with iBSP and HFS were assessed using the Mann–Whitney *U* test. A two-tailed *P*-value < 0.05 indicated statistical significance.

## Results

### Demographic information and clinical characteristics

The clinical and demographic characteristics of the 62 patients with iBSP, 62 with HFS, and 62 HCs are presented in Table 1. Both patient groups were similar to HCs in terms of age and gender. The iBSP and HFS groups did not differ in terms of the disease duration, JRS total scores, number of patients receiving BoNT injections, and duration of BoNT injections.

**TABLE 1:** Subjects’ demographics and clinical characteristics.

Groups	iBSP	HFS	HCs	*P*-values
Subjects	62	62	62	–
Median age, years (range)	55 (28–75)	53.5 (30–72)	57 (26–75)	0.472
Gender (male/female)	32/30	38/24	35/27	0.820
Median disease duration, years (range)	5 (0.5–25)	4 (0.08–20)	–	0.923
Median JRS total scores (range)	6 (2–8)	6 (0–8)	–	0.125
BoNT injections (yes/no)	48/14	41/21	–	0.231
Median BoNT injections duration, years (range)	3 (0.17–20)	2 (0.25–16)	–	0.554

BoNT, botulinum toxin; HCs, healthy controls; HFS, hemifacial spasm; iBSP, idiopathic blepharospasm; JRS, Jankovic Rating Scale.

### Between-group differences in network topological characteristics

**FIGURE 2:**
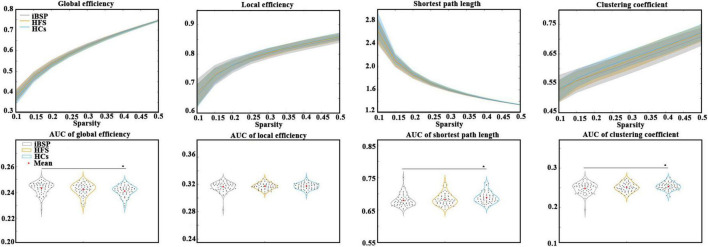
Comparisons of topological characteristics among groups. The top row shows the topological characteristics across a range of sparsity (0.1–0.5) at an interval of 0.05, and the bottom row shows the area under the curve of each topological characteristic. Group differences in network parameters were explored using analysis of covariance and *post-hoc* two-sample *t*-tests between any two groups, with age and gender as covariates. *Represents significant results with *P* < 0.05. AUC, area under the curve; HCs, healthy controls; HFS, hemifacial spasm; iBSP, idiopathic blepharospasm.

The E_glob_ of the cortico-basal ganglia networks was significantly increased, whereas the C_p_ and L_p_ of the cortico-basal ganglia networks were significantly decreased in patients with iBSP compared to HCs (Figure 2 and Table 2). No significant difference in global metrics was identified between the iBSP and HFS groups or between HFS groups and HCs.

**TABLE 2:** Differences of network parameters among three groups and their relationships with clinical measurements in patients with iBSP.

	Metric values (mean ± SD)			Group comparisons				Correlations	
	iBSP	HFS	HCs	iBSP-HFS-HCs	iBSP-HFS	iBSP-HCs	HFS-HCs	Duration (R, *P*)	JRS (R, *P*)
E_glob_	0.243 ± 0.004	0.242 ± 0.004	0.241 ± 0.004	0.016*	0.658	0.013*	0.306	(−0.240, 0.065)	(0.278, 0.031)*
C_p_	0.248 ± 0.013	0.251 ± 0.010	0.253 ± 0.009	0.042*	0.636	0.036*	0.607	(0.305, 0.018)*	(−0.265, 0.041)*
L_p_	0.679 ± 0.018	0.682 ± 0.017	0.688 ± 0.017	0.021*	1.000	0.021*	0.167	(0.230, 0.077)	(−0.272, 0.035)*

*Represents significant differences with P < 0.05. Group differences of network parameters were explored using analysis of covariance and post-hoc two-sample t-tests between any two groups with age and gender as covariates. Correlations between metric values and clinical measurements in iBSP were performed using partial correlation analyses with age and gender as covariates. C_p_, clustering coefficient; E_glob_, global efficiency; HCs, healthy controls; HFS, hemifacial spasm; iBSP, idiopathic blepharospasm; L_p_, shortest path length; SD, standard deviation. *Means significant results.

### Between-group functional connectivity differences

**FIGURE 3:**
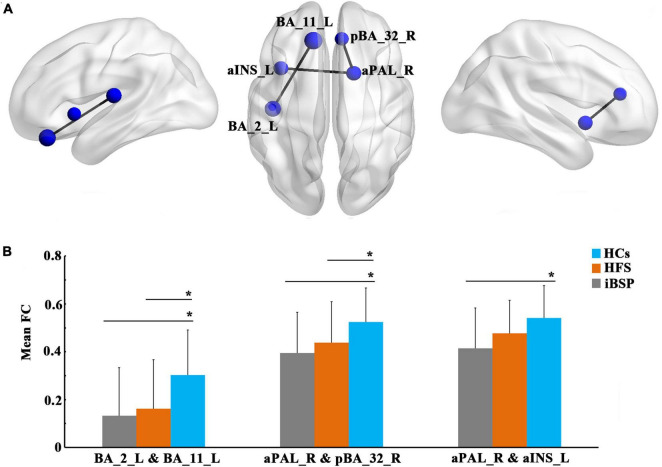
Decreased functional connections at the regional level. **(A)** Significantly decreased functional connections at the regional level in patients with idiopathic blepharospasm compared with healthy controls (HCs). The results were corrected by Bonferroni corrections of *P* < 1.22×10^– 5^. **(B)** Differences in mean functional connectivity among the three groups. Group differences in functional connectivity were explored using analysis of covariance and *post-hoc* two-sample *t*-tests between any two groups, with age and gender as covariates. *Represents significant results with *P* < 0.05. Abbreviations are listed in Table 3.

At the regional level, the functional connectivity between the left Brodmann area 11 (BA 11; orbitofrontal area) and left BA 2 (primary somatosensory cortex) and between the right anterior part of the pallidum (aPAL) and the right anterior part of BA 32 (dorsal anterior cingulate cortex) were significantly decreased in patients with iBSP and HFS compared to HCs (Figure 3 and Tables 3, 4) after Bonferroni corrections. No significant differences were found in these functional connections between iBSP and HFS groups. The functional connectivity between the right aPAL and the left anterior part of the insula (aINS) was lower in patients with iBSP, but not HFS, than HCs.

**TABLE 3:** The MNI coordinates of between-group functional connectivity differences of brain regions.

Abnormal brain regions	Full name	MNI coordinates (x, y, z)
BA_2_L	Left Brodmann area 2	(−43.4, −18.1, 12.1)
BA_11_L	Left Brodmann area 11	(−13.1, 35, −21.8)
aPAL_R	Right anterior part of pallidum	(17.1, 9.6, −6.6)
pBA_32_R	Right posterior part of Brodmann area 32	(8.1, 36.4, 16.1)
aINS_L	Left anterior part of insula	(−37.3, 13.5, −2.8)

MNI, Montreal Neurological Institute.

**TABLE 4:** Differences of resting-state functional connectivity among three groups and their relationships with clinical measurements in iBSP.

	Mean FC (mean ± SD)			Group comparisons				Correlations	
	iBSP	HFS	HCs	*F*-value	iBSP-HFS	iBSP-HCs	HFS-HCs	Duration (R, *P*)	JRS (R, *P*)
BA_2_L and BA_11_L	0.132 ± 0.200	0.161 ± 0.205	0.302 ± 0.188	< 0.001*	1	< 0.001*	< 0.001*	(−0.121, 0.362)	(0.005, 0.971)
aPAL_R and pBA_32_R	0.394 ± 0.170	0.437 ± 0.171	0.524 ± 0.142	< 0.001*	0.467	< 0.001*	0.007*	(0.036, 0.785)	(0.015, 0.911)
aPAL_R and aINS_L	0.414 ± 0.169	0.477 ± 0.137	0.540 ± 0.135	< 0.001*	0.049*	< 0.001*	0.058	(0.194, 0.141)	(−0.075, 0.571)

*Represents significant results with P < 0.05. Group differences of functional connectivity were explored using analysis of covariance and post-hoc two-sample t-tests between any two groups with age and gender as covariates. Correlations between functional connectivity and clinical measurements in iBSP were performed using partial correlation analyses with age and gender as covariates. aINS, anterior part of insula; aPAL, anterior part of pallidum; BA, Brodmann area; FC, functional connectivity; HCs, healthy controls; HFS, hemifacial spasm; iBSP, idiopathic blepharospasm; JRS, Jankovic Rating Scale; L, left; pBA, posterior part of Brodmann area; R, right; SD, standard deviation. *Means significant results.

### Between-group differences in regional indexes

No significant differences of regional indexes were found among three groups after Bonferroni corrections.

### Correlations between network properties and clinical characteristics

**FIGURE 4:**
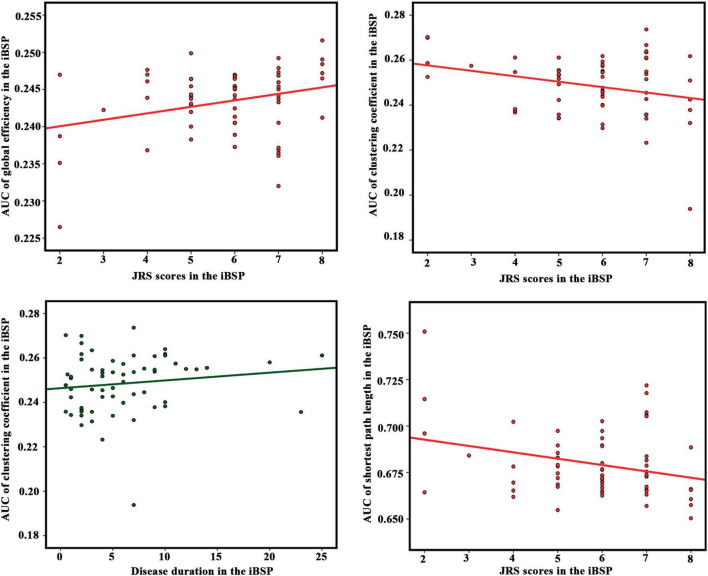
Correlation results between network parameters and clinical measurements in the idiopathic blepharospasm group. Partial correlation analyses were performed with age and gender as covariates. The significance level was set as *P* < 0.05. AUC, area under the curve; idiopathic blepharospasm (iBSP), idiopathic blepharospasm; JRS, Jankovic Rating Scale.

The E_glob_ of the cortico-basal ganglia networks was positively correlated with JRS total scores, whereas C_p_ and L_p_ were negatively correlated with JRS total scores in patients with iBSP. Moreover, the C_p_ of the cortico-basal ganglia networks was positively correlated with disease duration in patients with iBSP (Figure 4 and Table 2).

## Discussion

In this study, we found that patients with iBSP displayed alterations in integration and segregation in the functional cortico-basal ganglia networks and decreased functional connectivity in the orbitofrontal area, primary somatosensory cortex, anterior cingulate cortex, and pallidum compared with HCs. Current results support the hypothesis that extensively altered functional connections in the large-scale cortico-basal ganglia networks occur in patients with iBSP.

Considering that E_glob_ is inversely correlated with L_p_ between brain regions, our results (patients with iBSP displayed increased E_glob_ and decreased C_p_ and L_p_ compared with HCs) might suggest enhanced information transformation due to excessively optimized topological organization and global integration abilities in the cortico-basal ganglia networks in patients with iBSP. In addition, the existence of significant correlations between the severity of iBSP and graph theoretical metrics of the cortico-basal ganglia networks raises the possibility that abnormal topological organization in the cortico-basal ganglia networks is at least partly caused by excessive facial movements. Moreover, this possibility is further enhanced since these differences in E_glob_, C_p_, and L_p_ were not identified between iBSP and HFS groups. Thus, it is reasonable to speculate that facial hyperkinesia-related brain functional network changes in patients with iBSP might be attributed to the increase in facial activity and plastic changes in the brain white matter. This hypothesis is supported by a recent diffusion tensor imaging (DTI) study, which demonstrated that patients with iBSP exhibited increased network integration (significantly increased E_glob_ and decreased C_p_ and L_p_) in the whole-brain white matter networks (Guo et al., 2021), but such changes were no longer significant when patients with iBSP were compared to patients with HFS. The study also reported that patients with iBSP showed increased nodal efficiency in some regions located in the cortico-basal ganglia networks involved in sensorimotor, cognitive, and emotional processing, such as primary motor cortex, caudate nucleus, middle temporal regions, and posterior cingulate, and temporal thalamus. Moreover, these changes were not observed in patients with iBSP when compared with patients with HFS. In addition, plastic changes in multiple white matter tracts that mainly connect the basal ganglia, cortices, brainstem, and thalamus were reported in the iBSP group in another DTI study (Guo et al., 2020).

Interestingly, increased E_glob_ and decreased L_p_ of the cortico-basal ganglia networks were positively and negatively correlated with severity in patients with iBSP, respectively. Current evidence obtained from fMRI studies suggests that the basal ganglia circuit is involved in the triggering of spasms, whereas the cerebello-thalamo-cortical network plays a key role in symptom severity in the iBSP (Glickman et al., 2020). Moreover, the findings of DTI studies also suggest that the cerebellum or its pathways are involved in the intensity of dystonic spasms in the iBSP (Yang et al., 2014; Berman et al., 2018). In addition, Guo et al. (2021) did not find significant correlations of increased E_glob_ and decreased L_p_ in the whole-brain white matter networks with disease severity. They explained that the absence of infratentorial brain structures in the BNA-246 atlas they used may be responsible for it (Guo et al., 2021). The key brain regions that constitute the cerebello-thalamo-cortical network are partial components of the cortico-basal ganglia networks in our study. Whether the correlations between increased E_glob_ and decreased L_p_ in the functional cortico-basal ganglia networks and disease severity are mainly attributed to changes in the cerebello-thalamo-cortical network remains unclear; further studies are needed to address this question.

We also found that the functional connectivity between the left orbitofrontal area and left primary somatosensory cortex and between the aPAL and anterior part of the dorsal anterior cingulate cortex was significantly decreased in patients with iBSP and HFS compared to HCs. It has been shown that activation of the orbitofrontal area is involved in eye blinking (Tsubota et al., 1999). An fMRI study has also found that this area is associated with sensorimotor output (Yang et al., 2013). The primary somatosensory cortex, a vital component of the sensory system, has a significant role in somatosensory processing (Defazio et al., 2007). It has been proposed that focal dystonia should be considered a sensory system disorder characterized by defective somatosensory processing and impaired somatosensory capabilities (Tinazzi et al., 2000, 2009), suggesting that sensory dysfunction plays a key role in the pathophysiology of dystonia. Decreased connectivity of the primary somatosensory cortex with the orbitofrontal area in patients with iBSP and HFS might affect sensorimotor functional integration and indicate that iBSP and HFS may share overlapping pathophysiological mechanisms. The aPAL and anterior part of the dorsal anterior cingulate cortex is components of the limbic network. The aPAL is associated with behavior and cognitive performance without motor symptoms (Saad et al., 2020). The anterior cingulate cortex, particularly the dorsal portion, is involved in decision-making and working memory processes (Apps et al., 2016; Rolls, 2019). Previous studies demonstrated that patients with iBSP suffered from cognitive impairment (Alemán et al., 2009). Unfortunately, in the current study, we did not evaluate the cognitive function in patients with iBSP. Whether the abnormal functional connection between the aPAL and anterior part of the dorsal anterior cingulate cortex is related to impairments in non-motor (e.g., cognitive) aspects of iBSP requires further exploration.

This study has the following limitations. First, global signal regression in rs-fMRI remains to be debated; we did not perform this step for image pre-processing since global mean signal regression can lead to spurious resting-state functional correlations and false inferences. Second, some patients with iBSP or HFS were treated with BoNT injections; at present, the effects of BoNT on functional connections between regions in the cortico-basal ganglia networks at rest remain unclear. This limitation should be considered in further studies. Third, given the prevalence of depression, obsessive/compulsive symptoms, and cognitive dysfunction in specific cognitive domains in patients with iBSP, the associations between the dysfunction of cortico-basal ganglia networks and aforementioned non-motor symptoms in patients with iBSP are worth exploring in a future study. Finally, we did not find any difference in regional indexes between iBSP and HFS, or any correlations between these regional indexes and clinical measurements in the iBSP or HFS after Bonferroni corrections. Therefore, it is hard to determine which region was special for iBSP or HFS based on our current results.

In conclusion, we found a strong functional integration characterized by a high E_glob_ and a short L_p_ in the cortico-basal ganglia networks in patients with iBSP. Moreover, these network metrics were significantly correlated with disease severity of iBSP and might serve as quantitative markers for the evaluation of clinical symptom severity of iBSP.

## Data availability statement

The original contributions presented in this study are included in the article/Supplementary material, further inquiries can be directed to the corresponding authors.

## Ethics statement

The studies involving human participants were reviewed and approved by the Ethics Committee of the First Affiliated Hospital of Sun Yat-sen University. The patients/participants provided their written informed consent to participate in this study.

## Author contributions

QC: data analysis and drafting the manuscript. HX and YLu: data acquisition and drafting the manuscript. LZ, YG, YLi, AW, and ZO: data acquisition. XF and XZ: data analysis. WZ, HW, and QH: revising the manuscript. KP: design and revising the manuscript. JX: design, data analysis, drafting, and revising the manuscript. GL: design, data acquisition, drafting, and revising the manuscript. All authors contributed to the article and approved the submitted version.

## Footnotes

1
http://rfmri.org/dpabi

2
http://www.nitrc.org/projects/gretna/

## Supplementary material

The Supplementarya Material for this article can be found online at: https://www.frontiersin.org/articles/10.3389/fnins.2023.1159883/full#supplementary-material
