# Supplementary material for: Cortico-basal ganglia networks dysfunction associated with disease severity in patients with idiopathic blepharospasm
Source: Front Neurosci. 2023 Mar 30;17:1159883. doi: 10.3389/fnins.2023.1159883 (PMC10098005; doi:10.3389/fnins.2023.1159883)
Supplement: Supplementary file 1 [file Table_1.DOC]

**SUPPLEMENTARY MATERIAL**

**Cortico-basal ganglia networks dysfunction associated with disease severity in patients with idiopathic blepharospasm**

Qinxiu Cheng^1,†^, Han Xiao^2,†^, Yuhan Luo^3,†^, Linchang Zhong^4^, Yaomin Guo^3^, Xinxin Fan^1^, Xiaodong Zhang^1^, Ying Liu^3^, Ai Weng^3^, Zilin Ou^3^, Weixi Zhang^3^, Huawang Wu^5^, Qingmao Hu^1^, Kangqiang Peng^4^,*, Jinping Xu^1^,* and Gang Liu^3^,*

* These authors are corresponding authors.

^†^ These authors contributed equally to this work.

^1^Institute of Biomedical and Health Engineering, Shenzhen Institutes of Advanced Technology, Chinese Academy of Sciences, Shenzhen 518055, China

^2^Department of Nuclear Medicine, Guangdong Second Provincial General Hospital, Guangzhou 510310, China

^3^Department of Neurology, The First Affiliated Hospital, Sun Yat-sen University, Guangdong Provincial Key Laboratory for Diagnosis and Treatment of Major Neurological Diseases, National Key Clinical Department and Key Discipline of Neurology, Guangzhou 510080, China

^4^Department of Medical Imaging, Sun Yat-Sen University Cancer Center, State Key Laboratory of Oncology in Southern China, Collaborative Innovation Center for Cancer Medicine, Guangzhou 510060, China

^5^The Affiliated Brain Hospital of Guangzhou Medical University (Guangzhou Huiai Hospital), Guangzhou 510370, China

**Correspondence to:** Gang Liu,

Department of Neurology,

The First Affiliated Hospital, Sun Yat–Sen University, Guangdong Provincial Key Laboratory for Diagnosis and Treatment of Major Neurological Diseases, National Key Clinical Department and Key Discipline of Neurology, Guangzhou 510080, China

Phone: +86-20-87755766-8253

E-mail: liug26@mail.sysu.edu.cn

OR

Jinping Xu,

Institute of Biomedical and Health Engineering,

Shenzhen Institutes of Advanced Technology, Chinese Academy of Sciences, Shenzhen 518055, China

Phone: +86-755-86392226

E-mail: jp.xu@siat.ac.cn

OR

Kangqiang Peng,

Department of Medical Imaging,

Sun Yat-Sen University Cancer Center, State Key Laboratory of Oncology in Southern China, Collaborative Innovation Center for Cancer Medicine, Guangzhou 510060, China

Phone: +86-20-87342086

E-mail: pengkq@sysucc.org.cn

**Supplementary Table**

**Table S1** **Regions of interest (ROI) for the functional cortico-basal ganglia networks**

| **Region**  **number** | **Anatomical label** | **Hemisphere** | **MNI coordinates** | | |
| --- | --- | --- | --- | --- | --- |
|  |  |  | **X** | **Y** | **Z** |
| ROI_01 | BA 4 | R | 51.5 | -6 | 31 |
| ROI_02 | BA 4 | L | -36.3 | -16.4 | 41.6 |
| ROI_03 | BA 6 (SMA) | L | -1 | -0.5 | 54.4 |
| ROI_04 | BA 6 (premotor cortex) | R | 27.2 | 6.5 | 58 |
| ROI_05 | BA6 (premotor cortex) | L | -25.2 | 4.1 | 65.5 |
| ROI_06 | BA 6 ventral part | R | 8.1 | 0.7 | 71.9 |
| ROI_07 | BA 6 ventral part | L | -6 | 3 | 67.6 |
| ROI_08 | BA 1 | R | 59.5 | -17.6 | 44.8 |
| ROI_09 | BA 1 | L | -57.5 | -19.6 | 43.6 |
| ROI_10 | BA 2 | R | 39.3 | -22.4 | 16.2 |
| ROI_11 | BA 2 | L | -43.4 | -18.1 | 12.1 |
| ROI_12 | BA 3 | R | 37.3 | -42.4 | 44.6 |
| ROI_13 | BA 3 | L | -32.3 | -40.6 | 50.1 |
| ROI_14 | Insula posterior part | R | 42.4 | -3 | -2.6 |
| ROI_15 | Insula posterior part | L | -40.4 | -1.8 | -7.3 |
| ROI_16 | Posterior dorsal putamen | R | 36.3 | -8.2 | -0.5 |
| ROI_17 | Posterior dorsal putamen | L | -27.2 | -5.1 | -1.5 |
| ROI_18 | Posterior pallidum | R | 16.1 | 3.3 | -5.8 |
| ROI_19 | Posterior pallidum | L | -9 | 1.3 | -5.9 |
| ROI_20 | Thalamus posterior part | R | 12.1 | -30.1 | 2.8 |
| ROI_21 | Thalamus posterior part | L | -8 | -27 | 4 |
| ROI_22 | Cerebellum lobule 5 | R | 17.1 | -63.1 | -20.3 |
| ROI_23 | Cerebellum lobule 5 | L | -18.1 | -64.1 | -20.4 |
| ROI_24 | Cerebellum lobule 8 | L | -14.1 | -54.1 | -13.9 |
| ROI_25 | Cerebellum lobule 8 | R | 28.2 | -58 | -20 |
| ROI_26 | Cerebellar vermis | R | 1 | -73.8 | -35.3 |
| ROI_27 | BA 9 | R | 31.3 | 42.3 | 22.9 |
| ROI_28 | BA 9 | L | -30.3 | 34.9 | 26.9 |
| ROI_29 | BA 10 | R | 32.3 | 57.7 | 2 |
| ROI_30 | BA 10 | L | -37.3 | 49.8 | -5.5 |
| ROI_31 | BA 44 | R | 50.5 | 10.2 | 23.4 |
| ROI_32 | BA 44 | L | -38.3 | 5.2 | 21 |
| ROI_33 | BA 45 | R | 55.5 | 5.1 | 1.4 |
| ROI_34 | BA 45 | L | -53.5 | 3.1 | 0.2 |
| ROI_35 | BA 46 | R | 44.4 | 38.1 | 23.8 |
| ROI_36 | BA 46 | L | -40.4 | 35 | 24.7 |
| ROI_37 | BA 47 | R | 45.4 | 33.6 | -13.6 |
| ROI_38 | BA 47 | L | -47.4 | 33.6 | -12.4 |
| ROI_39 | BA 21 | R | 61.6 | -38.7 | -13 |
| ROI_40 | BA 21 | L | -61.6 | -29.5 | -10 |
| ROI_41 | BA 22 | R | 55.5 | -7.7 | -12.4 |
| ROI_42 | BA 22 | L | -51.5 | -12.7 | -16.2 |
| ROI_43 | BA 5 | R | 33.3 | -72.6 | 51.7 |
| ROI_44 | BA 5 | L | -32.3 | -67.6 | 54.1 |
| ROI_45 | BA 7 | R | 46.4 | -71 | 39.8 |
| ROI_46 | BA 7 | L | -44.4 | -67.9 | 37.8 |
| ROI_47 | BA 39 | R | 40.4 | -79.8 | 28.4 |
| ROI_48 | BA 39 | L | -32.3 | -86 | 29.2 |
| ROI_99 | BA 40 | R | 59.5 | -26.2 | 30.2 |
| ROI_50 | BA 40 | L | -59.5 | -28.4 | 32.2 |
| ROI_51 | BA 23 | R | 3 | -27.4 | 33.4 |
| ROI_52 | BA 31 | R | 5.1 | -32.8 | 38.5 |
| ROI_53 | BA 31 | L | -1 | -31.8 | 39.7 |
| ROI_54 | BA37 | R | 54.5 | -54.4 | -7.9 |
| ROI_55 | BA37 | L | -50.5 | -60.5 | -9.5 |
| ROI_56 | Caudate antero-dorsal | R | 12.1 | 13.2 | 4 |
| ROI_57 | Caudate antero-dorsal | L | -8 | 15.3 | 4 |
| ROI_58 | Putamen antero-dorsal | R | 28.2 | 4.8 | 7.9 |
| ROI_59 | Putamen antero-dorsal | L | -22.2 | 5.8 | 9 |
| ROI_60 | Thalamus anterior part | R | 9.1 | -5.6 | 9.5 |
| ROI_61 | Thalamus anterior part | L | -4 | -5.6 | 9.5 |
| ROI_62 | Thalamus medial part | R | 9.1 | -16 | 11.1 |
| ROI_63 | Thalamus medial part | L | -3 | -16.1 | 12.2 |
| ROI_64 | Cerebellum lobule 6 | R | 37.3 | -61 | -45.2 |
| ROI_65 | Cerebellum lobule 6 | L | -36.3 | -61.5 | -34.5 |
| ROI_66 | Cerebellum lobule 7 | R | 39.3 | -49 | -36.2 |
| ROI_67 | Cerebellum lobule 7 | L | -40.4 | -50 | -37.5 |
| ROI_68 | BA 11 | R | 17.1 | 33.9 | -20.7 |
| ROI_69 | BA 11 | L | -13.1 | 35 | -21.8 |
| ROI_70 | BA 25 | R | 2 | 19.2 | -13.2 |
| ROI_71 | BA 32 (posterior part) | R | 2 | 33.3 | 15.9 |
| ROI_72 | BA 32 (posterior part) | R | 8.1 | 36.4 | 16.1 |
| ROI_73 | BA 32 (subgenual part) | R | 0 | 34.2 | -2.8 |
| ROI_74 | BA 24 | R | 4 | 16.2 | 29.1 |
| ROI_75 | BA 38 | R | 56.5 | 11.9 | -13.6 |
| ROI_76 | BA 38 | L | -52.5 | 12.2 | -18.4 |
| ROI_77 | Insula anterior part | R | 32.3 | 14.8 | -7.5 |
| ROI_78 | Insula anterior part | L | -37.3 | 13.5 | -2.8 |
| ROI_79 | BA 35/36 | R | 27.2 | -32.2 | -18.5 |
| ROI_80 | BA 35/36 | L | -27.2 | -34.3 | -18.7 |
| ROI_81 | Hippocampus | R | 24.2 | -15.7 | -18.8 |
| ROI_82 | Hippocampus | L | -24.2 | -15.8 | -17.6 |
| ROI_83 | Amygdala | R | 24.2 | 5 | -21.2 |
| ROI_84 | Amygdala | L | -21.2 | 4 | -21.2 |
| ROI_85 | Ventral striatum | R | 10.1 | 10.7 | -8.9 |
| ROI_86 | Ventral striatum | L | -10.1 | 9.6 | -7.8 |
| ROI_87 | Anterior pallidum | R | 17.1 | 9.6 | -6.6 |
| ROI_88 | Anterior pallidum | L | -10.1 | 11.6 | -6.5 |
| ROI_89 | Thalamus medial line | R | 0 | -20.1 | 10.9 |
| ROI_90 | Thalamus ventral part | R | 10.1 | -22.6 | -2.5 |
| ROI_91 | Thalamus ventral part | L | -4 | -22.7 | -1.3 |

**BA**, Brodmann area; **L**, left; **MNI**, Montreal Neurological Institute; **R**, right; **ROI**, regions of interest; and **SMA**, supplementary motor area
